# Hemostatic Challenges in Neonates

**DOI:** 10.3389/fped.2021.627715

**Published:** 2021-03-02

**Authors:** Patricia Davenport, Martha Sola-Visner

**Affiliations:** Division of Newborn Medicine, Boston Children's Hospital, Boston, MA, United States

**Keywords:** neonate, bleeding, platelet function, hemostasis, platelet transfusion, FFP transfusion

## Abstract

The neonatal hemostatic system is strikingly different from that of adults. Among other differences, neonates exhibit hyporeactive platelets and decreased levels of coagulation factors, the latter translating into prolonged clotting times (PT and PTT). Since pre-term neonates have a high incidence of bleeding, particularly intraventricular hemorrhages, neonatologists frequently administer blood products (i.e., platelets and FFP) to non-bleeding neonates with low platelet counts or prolonged clotting times in an attempt to overcome these “deficiencies” and reduce bleeding risk. However, it has become increasingly clear that both the platelet hyporeactivity as well as the decreased coagulation factor levels are effectively counteracted by other factors in neonatal blood that promote hemostasis (i.e., high levels of vWF, high hematocrit and MCV, reduced levels of natural anticoagulants), resulting in a well-balanced neonatal hemostatic system, perhaps slightly tilted toward a prothrombotic phenotype. While life-saving in the presence of active major bleeding, the administration of platelets and/or FFP to non-bleeding neonates based on laboratory tests has not only failed to decrease bleeding, but has been associated with increased neonatal morbidity and mortality in the case of platelets. In this review, we will present a clinical overview of bleeding in neonates (incidence, sites, risk factors), followed by a description of the key developmental differences between neonates and adults in primary and secondary hemostasis. Next, we will review the clinical tests available for the evaluation of bleeding neonates and their limitations in the context of the developmentally unique neonatal hemostatic system, and will discuss current and emerging approaches to more accurately predict, evaluate and treat bleeding in neonates.

## Clinical Overview of Bleeding in the Neonate

Neonates, especially those born pre-term, are at high risk of bleeding, making this a commonly encountered problem in the Neonatal Intensive Care Unit (NICU). A recent study utilizing a standardized and validated neonatal bleeding assessment tool (NeoBAT) found that 25% of all neonates admitted to eight different NICUs experienced an episode of bleeding during their hospitalization, with 11% of episodes categorized as major/severe bleeds, 1% as moderate, and 13% as minor bleeds (1). In the same study, pre-term neonates <28 weeks' gestational age were found to have a higher incidence of bleeding compared to infants born at >28 weeks' gestation, highlighting the increased bleeding risk associated with a lower gestational age at birth.

This high incidence of bleeding, particularly among pre-term neonates, is at least partially related to factors specific to the neonatal population. These unique risk factors include the trans-placental passage of some hemostatically active vitamins (i.e., vitamin K) or maternal anti-platelet antibodies, and the developmental stage of blood vessels, the gastrointestinal tract, and the hemostatic system at varying gestational ages. In addition to these developmental stage-specific risk factors for bleeding, neonates are also at risk for more universal causes of bleeding due to their high incidence of sepsis, DIC, and frequent need for mechanical ventilation and critical care after birth. As such, the differential diagnosis for neonatal bleeding is broad and a thorough understanding of developmental stage, risk factors and underlying pathophysiology is critical to appropriately treat and try to prevent major bleeding in this vulnerable patient population. The goals of this review are to describe common clinical presentations of bleeding in neonates admitted to the Neonatal Intensive Care Unit (NICU), discuss the main developmental differences between neonatal and adult platelet function and hemostasis, review the tests available to evaluate and predict bleeding in neonates and their limitations, and evaluate current approaches to the management and prevention of clinically significant bleeding in infants. Of note, this review will not cover the hemostatic challenges of neonates on ECMO or neonates requiring cardiac surgery with cardiopulmonary bypass.

### Intraventricular Hemorrhage (IVH)

Intraventricular hemorrhage (IVH) is one of the most serious complications of prematurity owing to the critical window for brain development that occurs during fetal and neonatal life. IVH puts infants at risk for long term neurodevelopmental morbidity and mortality and, despite improvements in IVH rates over recent decades, its incidence remains high, affecting 15–25% of very- and extremely- premature infants (<32 and <28 weeks' gestational age, respectively) in the first week of life (2). Intraventricular hemorrhages often originate in a highly vascularized collection of neuronal-glial precursor cells called the germinal matrix (3). This region is selectively vulnerable to hemorrhage in premature infants due to its developmental paucity of pericytes, immature basal lamina, and deficiency of glial fibrillary acidic protein, all of which result in vascular fragility (3). When this fragile vasculature encounters disturbances in cerebral blood flow due to the impaired cerebral autoregulation of premature infants, hemorrhage can result. If the hemorrhage in the germinal matrix is substantial, the immature ependyma breaks and the cerebral ventricles fill with blood, becoming visible on head ultrasound evaluation (3). A number of antenatal, perinatal, and post-natal factors have been found to be associated with disturbances in cerebral blood flow and possibly IVH (most importantly gestational age), but most times the specific clinical circumstance or factor leading to hemorrhage remains unidentified. Currently, the only proven intervention to decrease the rate of IVH is the administration of antenatal steroids to the mother. A recent study demonstrated that administration of antenatal steroids decreased the rate of IVH by 16% in 24-25+6/7-week gestation infants, by 12.9% in the 26-27+6/7-week gestation infants, and by 19.4% across the entire cohort (4). Research is ongoing to find better strategies to prevent and manage this significant complication of prematurity.

### Pulmonary Hemorrhage

Pulmonary hemorrhage (PH) occurs in 3–5% of mechanically ventilated pre-term infants and in up to 8.6% of those born extremely premature (23–24 weeks' gestation) (5, 6). Additional risk factors include small for gestational age status, low Apgar scores, sepsis, presence of a patent ductus arteriosus (PDA), and severe respiratory distress syndrome (7). It most commonly occurs in the first 2 days of life and is associated with an increase in mortality overall and at 7 days of life (5). It has been hypothesized that PH occurs due to the rapid lowering of intrapulmonary pressure after administration of exogenous surfactant, which allows for an increase in pulmonary blood flow as blood shunts from the systemic to pulmonary circulation across the PDA (6). Currently, PH is managed with ventilator titration (increased positive end-expiratory pressure), administration of epinephrine via the endotracheal tube, and (after the acute phase) additional doses of surfactant. Platelet and/or FFP transfusions are frequently given to neonates with active PH, sometimes empirically in the setting of active bleeding and sometimes in response to decreased platelet counts or prolonged PT/PTT. However, the contribution of thrombocytopenia and/or coagulopathy to these hemorrhages remains uncertain.

### Lower Gastrointestinal (GI) Hemorrhage

Bloody stools can be seen in well-appearing newborns due to common causes such as swallowed maternal blood during delivery, the presence of an anal fissure, or allergic colitis. These cases are not associated with disorders of hemostasis, and typically resolve with close monitoring and the removal of cow's milk protein from the diet (in the case of allergic colitis). In contrast, in critically ill premature infants, frank rectal bleeding is most frequently seen in the setting of necrotizing enterocolitis (NEC). Necrotizing enterocolitis is one of the most common and devastating diseases in pre-term neonates with an estimated incidence of 7% among infants with a birth weight between 500 and 1,500 g (8) and an estimated incidence of death of 20–30%, with the highest mortality seen in those infants who require surgery (9). The typical presentation of NEC is feeding intolerance, abdominal distension, and bloody stools in a pre-term infant after 8–10 days of life, associated with bowel wall ischemia and bacterial overgrowth. These symptoms progress rapidly over hours often leading to systemic hypotension and respiratory failure, and can culminate with bowel perforation requiring surgery. Infants with severe disease also frequently develop severe thrombocytopenia ± coagulopathy associated with disseminated intravascular coagulation, further predisposing them to bleeding in the GI tract and other sites. Recent work in an animal model of NEC identified thrombin generation as an early event in the pathogenesis of NEC (10), which might trigger platelet activation as well as intravascular thrombosis. In this mouse model, thrombin inhibition as well as platelet depletion attenuated the severity of the intestinal injury and reduced mortality, pointing to a contribution of this pathway to the pathophysiology of the disease and highlighting potential mechanisms through which platelets could worsen the disease process.

### Minor Bleeding Events

In addition to the serious etiologies of neonatal bleeding mentioned above, neonates frequently experience minor bleeding events throughout their hospitalization. The presentations are numerous but include cephalohematoma sustained at birth, blood tinged endotracheal tube secretions in mechanically ventilated infants, and oozing from the umbilical stump or from sites of blood draws. It is unclear whether minor bleeding events are harbingers of more serious bleeding, but they often resolve with only close monitoring.

## Developmental Differences in Hemostasis

### Platelet Counts and Platelet Function in Neonates

The largest study on neonatal platelet counts conducted to date, which included ~47,000 infants delivered between 22 and 42 weeks' gestation (11), showed that platelet counts increased during gestation by ~2 × 10^9^/L per week. Importantly, the mean platelet count was ≥200 x 10^9^/L (well within the normal adult range) even in the most pre-term infants, but the 5th percentile was 104 × 10^9^/L for those ≤ 32 weeks gestation and 123 × 10^9^/L for late-pre-term and term neonates (11). This suggested that platelet counts between 100 and 150 × 10^9^/L might be more frequent among extremely pre-term infants than among full term neonates or older children/adults, and that perhaps different definitions of thrombocytopenia should be applied to neonates at different gestational ages. Nevertheless, even in the most pre-term neonates –just like in adults- platelet counts <100 x 10^9^/L are considered abnormal.

While platelet counts are similar in neonates and adults, there are substantial developmental differences in regard to platelet function. In *in vitro* platelet function assays, platelets from neonatal (full term) cord blood activate and aggregate less than adult platelets in response to traditional platelet agonists such as adenosine diphosphate (ADP), epinephrine, collagen, thrombin, and thromboxane analogs (12, 13). More recently, neonatal platelets were also found to exhibit a pronounced hyporesponsiveness to collagen-related peptide (CRP) and to the snake venom toxin rhodocytin, which activate the collagen receptor GPVI and the C-type lectin-like receptor 2 (CLEC-2), respectively (14). The few studies that have investigated platelet activation in pre-term neonates suggest that the platelet hyporeactivity might be more pronounced in premature infants compared to those born at term (13–15).

Different mechanisms contribute to the reduced responses of neonatal platelets to various agonists: 1. the hyporesponsiveness to epinephrine is due to a marked reduction in the number of α2-adrenergic receptors, the binding sites for epinephrine, on the surface of neonatal platelets; 2. the decreased responsiveness to thrombin is related to reduced expression of the thrombin receptors PAR-1 and PAR-4 in neonatal platelets (16); 3. the decreased response to thromboxane results from reduced signaling downstream from the receptor (17); 4. the reduced responses to collagen and rhodocytin result from mildly reduced expression of GPVI and CLEC-2, respectively, coupled with an intracellular signaling defect evidenced by reduced Syk and PLCγ2 phosphorylation following stimulation (14). Recently, developmental differences have also been described in regard to platelet *inhibitory* pathways, specifically a hypersensitivity of neonatal platelets to the inhibitory effects of prostaglandin E1 (PGE_1_) during ADP- and collagen-induced platelet aggregation, associated with a functional increase in the PGE_1_-cAMP-PKA axis (18).

Upon activation, in addition to experiencing a conformational change in the surface receptor GPIIb/IIIa that increases its affinity to fibrinogen, platelets secrete the content of their dense and alpha granules. In comparison with adult platelets, agonist-induced secretion of platelet granule content is reduced in both term and pre-term human neonates. In the case of dense granules, this might be related to the presence of fewer dense granules in neonatal compared to adult platelets (19). However, the 10-fold more abundant α-granules are present in similar numbers in neonatal and adult platelets (19–24). Recently, Caparros et al. demonstrated that the reduced exocytosis of alpha granules in neonatal platelets was associated with significantly reduced levels of syntaxin-11 and its regulator, Munc18b, which are SNARE proteins that mediate the fusion between vesicles and plasma membranes required for exocytosis (23). This provided a potential additional mechanism to explain the reduced degranulation of neonatal platelets in response to agonists.

**Figure 1:**
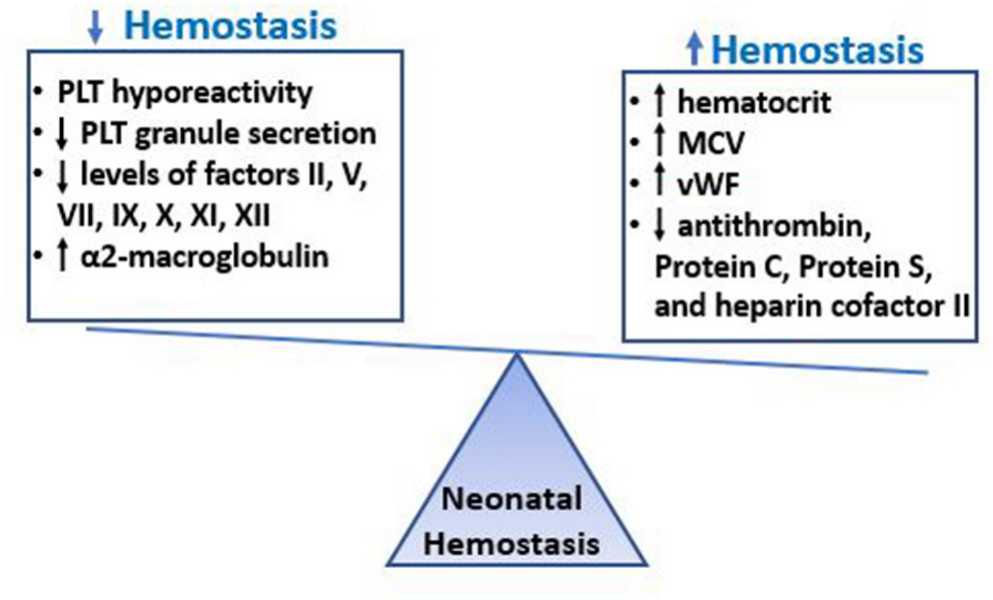
Neonatal Hemostasis. The hemostatic system of neonates is characterized by decreased platelet reactivity and decreased levels of multiple coagulation factors, which would predict a bleeding phenotype. However, these findings are exquisitely equilibrated by other factors in neonatal blood that promote clotting, such as the increased hematocrit, MCV, VWF levels and the low levels of natural anticoagulants. The overall hemostatic balance in healthy term neonates is different from that of adults but well-balanced, and perhaps slightly tilted toward thrombosis, with shorter bleeding times and faster initiation of coagulation compared to healthy adults.

Surprisingly, while the hypofunctional *in vitro* platelet phenotype would predict impaired primary hemostasis and a bleeding tendency, bleeding times (BTs) in healthy term neonates (using a device and technique modified to make a smaller skin incision) were found to be paradoxically ***shorter*** than BTs in adults (25). Similarly, studies using the Platelet Function Analyzer (PFA-100®, an *in vitro* test of primary hemostasis that measures the time it takes to occlude a small aperture, or Closure Time) found that cord blood samples from term neonates exhibited ***shorter*** closure times (CTs) than blood samples from older children or adults (26–29). These paradoxical findings, in the setting of platelet hyporeactivity, are explained by the presence of multiple factors in the blood of healthy neonates that enhance platelet/vessel wall interactions, including higher concentrations of circulating vWF with enhanced adhesive activity due to a predominance of ultralong polymers (30–33), higher hematocrits, and a higher MCV (34). These factors effectively counteract the platelet hyporeactivity, with the net effect of shorter bleeding times in neonates compared to adults (Figure 1). In support of the importance of the hematocrit on neonatal primary hemostasis, hematocrits below 28% in pre-term neonates were associated with longer bleeding times, which improved following red cell transfusion (35).

These compensatory mechanisms might be less well-developed in pre-term infants, whose platelets are also more hyporeactive than those of full-term infants, potentially leading to a more vulnerable primary hemostatic system. In fact, BTs performed on the first day of life were longer in pre-term compared with term infants, with neonates <33 weeks gestation exhibiting the longest BTs (36). PFA-100 CTs from non-thrombocytopenic neonates were also inversely correlated to gestational age in both cord blood and neonatal peripheral blood samples obtained on the first day of life (37). However, CTs in pre-term neonates were still near or within the normal range for adults, suggesting that healthy pre-term neonates also have adequate primary hemostasis.

Neonatal platelet function, measured as platelet activation by flow cytometry, improves significantly post-natally and nearly normalizes by 10–14 days, in term as well as pre-term infants (13, 15). Consistently, Del Vecchio et al. found that all infants had shorter BTs by day of life 10 than at birth, and that early gestational age-related differences disappeared by then. Moreover, little or no further shortening occurred between days 10 and 30 (36). PFA-100 CTs are also significantly longer in the blood of neonates as early as on day of life 1–2 compared to cord blood, but remain similar to or shorter than those of adults (37).

Taken together, while the decreased platelet function of neonates has been invoked as a potential contributor to the high incidence of bleeding among neonates, particularly those born pre-term, the evidence suggests that–under normal circumstances- this platelet hyporeactivity is not a developmental defect, but rather an integral part of a developmentally distinct, but well-balanced neonatal primary hemostatic system. Key points related to neonatal primary hemostasis are summarized in Table 1.

**Table 1:** Key points regarding neonatal primary hemostasis and platelet transfusions.

**Platelet count and function**	• Mean platelet counts are within the normal adult range even in the most pre-term neonates • Neonatal platelets are hyporeactive in response to most agonists *in vitro* (pre-term > term) • Platelet reactivity improves to near-adult levels by day of life 10–14
**Compensatory factors**	This platelet hyporeactivity is balanced by factors in neonatal blood that enhance platelet/vascular wall interaction: • Increased hematocrit • Increased MCV • Increased vWF and predominance of ultralong multimers
**Tests of primary hemostasis**	• Bleeding times are shorter in healthy neonates than in adults • PFA-100 closure times are shorter in term cord blood samples than in adult blood samples. • These suggest enhanced primary hemostasis in neonates, despite their platelet hyporeactivity.
**Effects of platelet transfusions**	• Platelet transfusions should be administered to thrombocytopenic neonates *with active bleeding* • Non-bleeding pre-term neonates who received prophylactic platelet transfusions for PC <50 × 10^9^/L had significantly *higher* bleeding and mortality compared to neonates transfused only for PC <25 × 10^9^/L. • High risk, critically ill neonates benefit from the lower transfusion threshold as much as low risk stable neonates.

Ontogenetically, these differences in platelet reactivity might be an important mechanism to prevent unwanted platelet activation and thrombosis during birth, a process frequently associated with tissue injury and epinephrine surges. However, how disease processes perturb this delicate system, and whether these disturbances contribute to bleeding, are unanswered questions.

### The Neonatal Hemostatic System

There are also substantial differences between the fetal/neonatal and the adult coagulation system. In the classical coagulation cascade model, clotting factors interact and undergo a series of enzymatic reactions via the contact activation (intrinsic) and tissue factor (extrinsic) pathways, which converge on a final common pathway that culminates in thrombin formation. Coagulation factors do not cross the placenta because of their size, and are produced in the fetus starting at 11 weeks gestation. The levels of most coagulation factors increase during gestation and post-natally, and therefore are lower in pre-term compared to term neonates, and in term neonates compared to older children and adults. In full term infants, levels of most coagulation factors (particularly the vitamin K-dependent ones) are ~50% of the adult levels, and increase to near-adult values by 6 months of age (38, 39). The activity of vitamin K-dependent factors is further reduced in pre-term infants, to ~30% in 24–29 weeks' gestation infants. In contrast to vitamin K-dependent factors, neonates have normal levels of factor VIII, factor XIII and fibrinogen, and elevated levels of vWF, as described above (40). Keeping in mind these developmental differences in the activity levels of the different coagulation factors is essential to adequately interpret laboratory results in neonates.

The PT and PTT, the two most commonly used tests to evaluate coagulation status, are measures of the intrinsic and the extrinsic clotting pathways, respectively. Both tests were developed specifically to investigate coagulation factor deficiencies, and thus are longer in healthy pre-term and term neonates compared to adults, reflecting the lower activity levels of most coagulation factors in these populations. Andrew *et al*. published the first PT, PTT and fibrinogen reference ranges for healthy near-term and term infants in 1987 (30), followed by a study in pre-term neonates 30–36 weeks in 1988 (31). These studies included reference ranges for infants on days of life 1, 5, 30, 90, and 180, which showed rapid changes in the first few days after birth and through infancy. In a subsequent study, the same group measured the concentration of 33 components of the hemostatic system in children ages 1 to 16 and showed important physiologic differences between the hemostatic system of children and that of adults (41). Taken together, these observations led to the concept of “developmental hemostasis” to describe a series of age-related physiological changes of the coagulation system from fetal to neonatal to pediatric and ultimately to adult life.

Focusing on more pre-term infants, Christensen et al. published reference ranges for infants <34 weeks' gestation, using cord blood samples (42), and Neary et al. reported PT, PTT and fibrinogen reference ranges obtained on day of life 1 in a cohort of 183 infants born at <27 weeks' gestation, the group at highest risk of clinically significant bleeding. In this high-risk cohort, the median (range) PT was 20.2 (14.4–36.7) s, PTT was 67.4 (34.9–191.6) s and fibrinogen 1.4 (0.5–4.8) g/L (43). These values were higher than those reported by Christensen et al., which could have been related to the source of the blood (cord blood vs. neonatal blood) and/or to the use of different reagents and testing systems (44). Taken together, these studies indicated that the upper limits for both PT and PTT values are higher among healthy extremely pre-term neonates than among moderately pre-term or term infants (Table 2), consistent with the developmental differences in clotting factor levels. However, both PT and PTT decrease rapidly in the first few days after birth, with significantly lower levels noted on day of life 3 compared to day of life 1 in extremely pre-term neonates (45).

**Table 2:** Neonatal reference ranges for common coagulation tests measured on day of life 1, by gestational age.

	**PT (s)**	**PTT (s)**	**Fibrinogen (mg/dL)**
<27 weeks^*^	14.4–36.7	40.5–158.5	70–480
28–34 weeks^**^	13.9–20.6	30–57	87–470
30–36 weeks^*^	10.6–16.2	27.5–79.4	150–373
Full Term^***^	10.1–15.9	31.3–54.5	167–399

* *Reference ranges reflect 2.5th−97.5th percentiles (31, 43)*.

** *Reference ranges reflect 5th−95th percentiles (42)*.

*** *Reference ranges calculated from mean ±SD (2SD below and above the mean) (30)*.

In 2006, Monagle et al. published comprehensive reference ranges for coagulation tests measured in healthy term neonates on days of life 1 and 3 and in children ranging from 1 month to 16 years, using more modern coagulation testing systems (44), and confirmed the concept of developmental hemostasis initially described by Andrew et al. The physiologic changes in the coagulation system over the course of development and their implications for clinical practice have been described in detail in recent reviews (39, 46). However, it is important to keep in mind that the actual values of the various tests vary depending on the reagents and analyzer system utilized, in addition to the patient's age (44). Thus, individual coagulation laboratories need to develop age-related reference ranges using their own testing systems, in order to interpret the results adequately.

The “prolongation” of the PT and PTT in neonates and particularly in extremely pre-term neonates, who have the highest incidence of major bleeding, has been frequently interpreted as a hemostatic defect and has led to the practice in many Neonatal Intensive Care Units (NICUs) of routinely checking coagulation tests and administering FFP to non-bleeding pre-term neonates with “abnormal” values to try to prevent bleeding, particularly IVH (43, 47). Paradoxically, however, studies by Cvirn et al. using assays with physiologic amounts of tissue factor found adequate and *faster* thrombin generation (the final product of the coagulation cascade) in neonates compared to adults (48). More recently, Neary et al. found that thrombin generation was similar in very pre-term compared to term cord blood samples, despite differences in PT and PTT levels. Interestingly, lag time and time to peak thrombin generation were ***shorter*** in pre-term compared to term cord blood samples, indicating *faster* thrombin generation in the pre-term infants (45).

While the reasons for the discrepancy between prolonged coagulation times and adequate/faster thrombin generation in pre-term compared to term infants (and in neonates compared to adults) are not fully understood, they likely involve the co-existent developmental differences in *anticoagulant* pathways in neonates, which are not well-reflected in the PT and PTT assays. Indeed, just like most coagulation factors are decreased in neonates, most natural anticoagulants are also reduced at birth (30, 31, 41), which results in a balanced neonatal hemostatic system. Specifically, antithrombin (AT), heparin cofactor II and proteins C and S levels are significantly reduced in neonates (both term and pre-term), ~50% of adult levels at birth (39). Levels of these anti-coagulants increase slowly after birth, and AT and heparin cofactor II reach adult levels by 3 months of age (41). Perhaps to compensate for these low levels, another anticoagulant, α2-macroglobulin, is present at higher levels in neonates than in adults, and further increases over the first 6 months of age (30, 41). Taken together, the evidence suggests that the prolonged PT and PTT found in otherwise healthy pre-term and term neonates should not be interpreted as a developmental deficiency or a bleeding tendency, but rather as a limitation of these tests to reflect the complexities of a developmentally unique but well-balanced neonatal hemostatic system (Figure 1 and Table 3).

**Table 3:** Key points regarding neonatal secondary hemostasis and FFP transfusions.

**Coagulation factor levels**	• Neonates have reduced levels of most coagulation factors, particularly vitamin K-dependent factors • FVIII, FXIII, and fibrinogen levels are normal • vWF levels are elevated • Coagulation factor levels change in neonatal life, infancy and childhood following specific patterns (developmental hemostasis).
**Natural anticoagulant levels**	• Neonates have low levels of AT, HCII, protein C and protein S • α2-macroglobulin is present at higher levels in neonates than in adults
**Laboratory evaluation of hemostasis**	• PT and PTT are longer in healthy neonates than in healthy adults (pre-term > term) and decrease in the first few days after birth • Actual values for tests of coagulation vary depending on the source of the sample (cord blood vs. neonatal blood), reagents and testing systems used. • However, thrombin generation is faster in neonates than in adults (pre-term > term) • Tests of whole blood hemostasis (TEG, ROTEM) show faster initiation and propagation of coagulation in neonates compared to adults
**FFP transfusions**	• FFP should be administered to neonates who present with bleeding associated with coagulation factor(s) deficiency, if the specific factor is not known or available. • Prophylactic FFP transfusions given empirically to pre-term neonates or in response to “prolonged” PT or PTT do not decrease the incidence or severity of IVH.

*vWF, von Willebrand Factor; AT, antithrombin; HCII, Heparin cofactor II; IVH, Intraventricular hemorrhage*.

## Evaluation of Bleeding and Prediction of Bleeding Risk in Neonates

### Platelet Counts

Platelets are essential for primary hemostasis. For that reason, a platelet count is always part of the initial evaluation of a neonate who presents with abnormal bleeding. In that setting, the finding of thrombocytopenia (either isolated or as part of a coagulopathy) is an important diagnostic clue. Severe isolated thrombocytopenia in an otherwise healthy neonate should raise suspicion for Fetal/Neonatal Alloimmune Thrombocytopenia (caused by the transplacental passage of maternal alloantibodies directed against antigens in the fetal platelets) which is associated with an incidence of bleeding of 10–20% (49, 50). Isolated thrombocytopenia can also be found in neonates with a history of intrauterine growth restriction or with viral, bacterial or fungal infections. In critically ill neonates with bleeding in the setting of sepsis, NEC, or severe perinatal asphyxia, thrombocytopenia is frequently present, either in isolation or as part of a picture of disseminated intravascular coagulation (DIC).

The utility of the platelet count as a predictor of bleeding risk in neonates *without* abnormal bleeding is much more controversial, although in neonatal clinical practice thrombocytopenia has been widely considered a risk factor for bleeding, particularly among pre-term infants. In support of an association between thrombocytopenia and bleeding, a recent study of 972 very-low-birth-weight infants (VLBW, <1,500 g at birth) found that having a platelet count <150 × 10^9^/L in the first week of life was associated with a higher incidence of IVH (hazard ratio 2.17; 95% CI, 1.53–3.08; *p* < 0.001). However, association does not imply causality, and the same study found no correlation between the severity of thrombocytopenia and the risk for IVH (51). The latter finding, consistent with several other studies (52–56), raised serious questions regarding the value of the platelet count as a marker of bleeding risk in pre-term neonates, and the effectiveness of platelet transfusions at preventing hemorrhage in non-bleeding neonates with mild to moderate thrombocytopenia (see Platelet Transfusions below). Interestingly, the same lack of correlation between severity of thrombocytopenia and significant bleeding has been reported in pediatric patients with chemotherapy-induced thrombocytopenia (57), suggesting that factors other than the platelet count might be the main determinants of bleeding risk in thrombocytopenic children as well as neonates.

Recently, other approaches have been suggested to assess bleeding risk in thrombocytopenic pre-term neonates. In a study evaluating platelet function in pre-term and term neonates by flow cytometry, fibrinogen binding and degranulation responses to ADP were significantly reduced in septic compared to healthy neonates, raising the possibility that tests of platelet function might eventually contribute to identify neonates at high risk of bleeding (58). However, whether these platelet functional differences are associated with a higher incidence or severity of bleeding remains to be determined. Given the unique features and dynamic nature of the neonatal primary hemostatic system, we hypothesized that a whole blood test of primary hemostasis, such as the closure time in response to collagen and ADP (CT-ADP) measured in the Platelet Function Analyzer-100 (PFA-100), would be a better marker of bleeding risk in pre-term neonates than the platelet count or the platelet function alone, since it would measure the combined effects of platelet count, platelet function, hematocrit, and vWF levels on a baby's primary hemostatic ability. Indeed, in a cohort of 54 infants with gestational age <27 weeks, we found a significant correlation between median CT-ADP (but not platelet count) and bleeding severity, quantified using a validated neonatal Bleeding Assessment Tool (55). Furthermore, changes in the CT-ADP were strongly correlated with changes in the bleeding score, while changes in platelet counts were not (59). This study suggested that tests of whole blood primary hemostasis might be more useful markers of bleeding risk among thrombocytopenic pre-term neonates than platelet counts, although the relatively high volume of blood required for the CT-ADP (800 μL) precludes its current widespread use in this population. Finally, Fustolo-Gunnink and collaborators developed a dynamic model to predict major bleeding in pre-term neonates at any time-point during the first week after the onset of severe thrombocytopenia, which incorporated the variables gestational age, post-natal age, intrauterine growth retardation, NEC, sepsis, platelet count and mechanical ventilation (60). While not yet prospectively validated, this is a promising approach to making individualized treatment decisions in this population and has the unique advantage of incorporating *in vivo* risk factors that cannot be captured by any laboratory test.

### PT/PTT

As previously described, the PT and PTT can be prolonged in neonates at baseline, especially in those born premature, likely reflecting an aspect of the developmentally unique neonatal hemostatic system. Thus, it is difficult to decide how to interpret and respond to these values in a non-bleeding neonate, and studies have shown no association between results of these tests and IVH rates in pre-term neonates (42, 45). However, when faced with neonatal bleeding of unclear etiology, the PT and PTT can be very useful to evaluate for specific coagulation factor deficiencies, which can present with clinical bleeding and a prolongation of either the PT or PTT beyond that seen in healthy neonates (Table 4).

**Table 4:** Typical test result patterns in bleeding disorders that can present in the neonatal period.

**Disease**	**PT**	**PTT**	**Platelets**
Hemophilia A	Normal	↑↑↑	Normal
Hemophilia B	Normal	↑↑↑	Normal
Factor XIII Deficiency	Normal	Normal	Normal
Vitamin K Deficiency of the Newborn	↑↑	Normal	Normal
vWD type 3	Normal	↑↑↑	Normal
DIC	↑↑	↑↑	↓↓↓

*vWD, Von Willebrand Disease; DIC, Disseminated Intravascular Coagulation*.

#### Vitamin K Deficiency of the Newborn (Previously Hemorrhagic Disease of the Newborn)

Clinically, infants suffering from Vit K deficiency present with visible frank bleeding from the nose, umbilicus, skin, urinary tract, GI tract, and from sites of needle pricks. Less visible, but more devastating, they also can suffer from intracranial, pulmonary, and massive GI bleeding (61). Vit K deficiency leads to a reduction in the activity of the Vit K-dependent coagulation factors II, VII, IX, X, and of the anticoagulant protein C and protein S. It should be suspected in a neonate who presents with abnormal bleeding and a marked prolongation of the PT, which measures the activity of three of the four Vit K-dependent factors (II, VII, and X) (61). Vit K deficiency has been classified into early, classical, and late disease, each with unique etiologies and presentations (Table 5). Early Vit K deficiency is rare and presents in the first 24 h of life in an infant whose mother took medications during pregnancy that interfere with Vit K metabolism (such as warfarin, phenytoin, rifampin, and isoniazid). Classical Vit K deficiency presents between 2 and 7 days of birth and is likely due to inadequate oral feeding, given how critical the successful establishment of breast feeding is to Vit K status (62). Late Vit K deficiency presents between 8 days and 6 months of life. This form nearly always occurs in exclusively breast-fed infants and has a higher incidence of intracranial hemorrhage, which is often the presenting sign (63). Many of the affected infants have hepatobiliary dysfunction, resulting in cholestasis and impaired secretion of bile salts that lead to malabsorption of Vit K (64). With the nearly universal administration of intramuscular Vit K after birth, the incidence of Vit K deficiency (in particular the classical form) markedly decreased. However, the increasingly frequent refusal to Vit K administration among parents in westernized countries has led to a trend of increasing cases. Some healthcare systems or families opt for a multi-dose oral regimen of Vitamin K after birth, but this is not recommended due to concerns for poor enteral absorption and compliance. Neonates who present with acute bleeding due to Vit K deficiency should be treated upon suspicion with intravenous Vit K, which will reverse the coagulopathy (62). However, due to the time required for Vit K to take effect, FFP should also be administered immediately to prevent devastating intracranial hemorrhage.

**Table 5:** Classification of vitamin K deficiency of the newborn.

	**Risk factors**	**Clinical presentation**
Early (24 h of life)	Maternal medications: • Vitamin K antagonists • Anticonvulsants • Tuberculosis drugs	• Umbilical stump bleeding • Cephalohematoma • ICH
Classical (1–7 days of life)	Inadequate Vitamin K due to: • Lack of prophylaxis • Poor breastfeeding	• GI bleeding • Mucocutaneous bleeding • Oozing at umbilicus or circumcision site • ICH
Late (8 days to 6 months of life)	• Exclusive breastfeeding • Poor feeding • GI disorders • Liver disease • Pancreatic disease	• GI bleeding • Mucocutaneus bleeding • Very high risk for ICH • Death

*ICH, Intracranial hemorrhage; GI, Gastrointestinal*.

#### Hemophilia A and B

Hemophilia A and B, caused by deficiencies of Factor VIII and Factor IX, respectively, are the most common inherited bleeding disorders that present in neonatal life. They are classically inherited in a X-linked recessive pattern, but ~1/3 of cases are due to spontaneous genetic mutations with no family history. The diagnosis of hemophilia is occurring at earlier ages, with over half of cases now being diagnosed in the neonatal period (65). Reasons prompting testing and leading to the diagnosis in newborn infants are listed in Table 6. Unlike older children with hemophilia, who present with hemarthroses, neonates typically present with iatrogenic bleeding (oozing or excessive hematoma formation after venipuncture or intramuscular Vit K administration), excessive bleeding after circumcision, or intracranial/extracranial bleeding (66–68). In a survey of 102 neonates with cranial bleeds and hemophilia, the mean age at diagnosis was 4.5 days, and intracranial hemorrhages (most frequently subdural) were more common than extracranial bleeds (such as cephalohematomas and subgaleal hemorrhages) (69). A clinical suspicion of hemophilia is supported by an isolated prolongation of the PTT, but definitive diagnosis requires measurement of Factor VIII or IX. Since factor VIII levels are within the normal adult range in both pre-term and term neonates, it is possible to diagnose hemophilia A of any severity in the neonatal period. However, this is not true for Factor IX, which shows reduced levels at birth and an even further reduction in infants born pre-term. Thus, severe hemophilia B can be diagnosed at birth, but confirmation of mild cases requires repeat testing at 6 months of age or genetic analysis (if a familial genetic defect is known). The delivery room management of an infant with known or suspected hemophilia has been the subject of multiple retrospective studies (69–72). Current recommendations state that there is no contraindication to a vaginal delivery, but an instrumented delivery (i.e., forceps, vacuum extraction, and the use of scalp electrodes) should be avoided and early transition to cesarean delivery is recommended if there is a failure of labor (73, 74).

**Table 6:** Reasons for diagnostic testing in newborns with hemophilia^*^.

Number of infants ages 0–2 years	864
Number diagnosed within 1 month of birth	633 (73%)
Reason for diagnosis	
Carrier mother	299 (47.2%)
Other family history	147 (23.2%)
Bleeding event	182 (28.8%)
Unknown	5 (0.8%)

* *Data from the Universal Data Collection (UDC) 2010 update (65)*.

#### Isolated Coagulation Factor Deficiencies

Outside of hemophilia A and B, neonates can inherit deficiencies of other isolated coagulation factors (75). Von Willebrand disease (VWD) is the most frequent inherited bleeding disorder, affecting ~1% of the population. It is classified into three categories based on the quantitative level or function of von Willebrand Factor (vWF). Type I is due to a quantitative deficiency of vWF and typically has a mild presentation with mucosal bleeding. Type II is due to a qualitative defect in vWF and is divided into four subtypes, which are associated with more severe bleeding phenotypes than Type I. Due to the increased levels of vWF and the presence of high molecular weight vWF multimers in neonates, these types do not typically present in neonatal life. However, type III VWD is the most severe form, due to a complete or almost complete deficiency of vWF, and this form can present in neonates with a phenotype similar to severe hemophilia A (76).

Other factor deficiencies can present in the neonatal period but are rare and diagnosis requires an astute clinician with a high index of suspicion and often assistance by a pediatric hematologist. Deficiency of Factor XIII, which is responsible for cross linking fibrin and stabilizing clots, classically presents with delayed umbilical cord hemorrhage but a normal PT and PTT. Thus, the diagnosis requires a high index of suspicion prompting the measurement of Factor XIII levels. Alpha2-antiplasmin and plasminogen activator inhibitor-1 both act to reduce plasmin activity and deficiency of either is extremely rare, but should also be considered when the PT and PTT are normal in a neonate with abnormal bleeding (76). In afibrinogenemia and in Factor II, V, and X deficiency the PT and PTT are both prolonged (76–78). Finally, Factor VII deficiency is a rare, heritable bleeding disorder with variable presentation and over 250 causal mutations (79). Neonates often present with multifocal spontaneous bleeding in the first few days of life that can range from epistaxis, gum bleeding and hematomas to hemarthrosis and life-threatening cerebral and gastrointestinal hemorrhages (80). Coagulation studies reveal a prolonged PT, and the diagnosis is confirmed by low Factor VII levels (75).

### Tests of Global Hemostasis: TEG and ROTEM

Thromboelastography (TEG) and Rotating Thromboelastometry (ROTEM) are both viscoelastic point-of-care tests that offer rapid global assessments of whole blood hemostasis in small volumes of whole blood, making them ideal tests for neonatal patients with small blood volumes. Both assays provide information on platelet function, clot formation, tensile strength of the clot, and subsequent clot lysis. While similar, the values obtained from TEG and ROTEM assays are not interchangeable, but both can help guide the selection of blood products for transfusion in a bleeding patient. Given the developmental differences in neonatal hemostasis suggested by standard coagulation tests (PT/PTT), investigators have been interested in comparing measures of global hemostasis between neonates and adults using these assays. Multiple studies have found contrasting results, likely due to differences in sample collection (umbilical cord blood vs. peripheral venous or arterial blood) and in anticoagulant used. Initial small studies using the TEG assay in neonates did not demonstrate differences in fibrin clot formation, clot strength, or rate of fibrinolysis (81, 82), but more recent studies found that neonates have *faster* initiation and propagation of coagulation (83–86), consistent with the faster thrombin generation described above. This relative pro-coagulant state seen on TEG, despite prolongation of conventional coagulation tests, reinforces the theory that neonatal hemostasis is not defective, but rather carefully balanced in a developmental stage-specific manner. A study comparing ROTEM values in pre-term vs. full-term infants found that maximal clot firmness (MCF) was significantly lower in pre-term compared to full-term neonates (87). However, this and a subsequent study (84) found no association between any TEG parameter and the occurrence of post-natal complications in pre-term infants, including intraventricular hemorrhage (IVH). Conversely, a recent study comparing values of healthy neonates to those of bleeding neonates at different gestational ages found statistically significant differences in assay values that were associated with clinical bleeding (86). This study also described reference ranges for both citrate-modified and heparinase-modified TEG values, given the frequency of these modifications in neonatal blood samples.

While TEG has been studied more extensively in neonates, ROTEM has only recently begun to be used in this population. One study described ROTEM references ranges in a pediatric cohort from 0 months to 16 years and found differences among all age groups (83). The most striking differences were with infants 0–3 months of age, who exhibited accelerated initiation and propagation of coagulation and increased clot firmness despite prolonged standard plasma coagulation tests (PT/PTT) (83). As reference ranges have become published, investigators have started assessing the utility of the ROTEM to evaluate coagulation in common neonatal clinical conditions, such as sepsis and congenital heart disease (CHD). Initially, one group found that a hypercoagulable ROTEM profile on the first day of neonatal sepsis was associated with disease severity (88). In a more recent study, the same group found that septic neonates were more likely to show fibrinolysis shutdown on ROTEM than non-septic neonates, but the test could not discriminate septic from non-septic neonates and could not predict clinical outcomes (89). It has also been well-described that neonates with CHD often have coagulation abnormalities that result in an increased incidence of bleeding (90, 91). One study evaluated hemostasis in these infants with ROTEM and found that neonates with cyanosis secondary to CHD had inferior clot formation as well as a higher incidence of abnormal parameters when compared to non-cyanotic CHD patients and healthy controls (92), suggesting that cyanosis and/or polycythemia may have an impact on hemostasis.

## Interventions to Manage and Prevent Bleeding in Neonates

### Platelet Transfusions

Platelets are essential for hemostasis and contribute substantially to clot formation. For that reason, in the setting of acute bleeding, neonates are frequently transfused platelets if the platelet count is <100 × 10^9^/L, or empirically in cases of life-threatening acute hemorrhage (i.e., as part of a massive transfusion protocol). However, the majority of platelet transfusions in the NICU are given to non-bleeding neonates, when the platelet count falls below an arbitrary level below which the risk of bleeding is thought to increase.

Historically, it has been widely accepted that thrombocytopenic pre-term neonates should receive platelet transfusions at higher platelet count (PC) thresholds than older children and adults due to their high incidence of spontaneous intracranial bleeding, particularly intraventricular hemorrhage (IVH). Over the last decade, several surveys and observational studies revealed a striking world-wide variability in neonatal platelet transfusion thresholds, and an overall more liberal approach to platelet transfusions in U.S. compared to European NICUs (93, 94). This variability was at least in part due to the paucity of high-level evidence in the field. Until recently, there was only one randomized trial of platelet transfusion thresholds in pre-term neonates, published 25 years ago (95). That study randomized 152 Very-Low-Birth-Weight (VLBW, <1,500 g at birth) neonates to receive platelet transfusions for platelet counts <150 × 10^9^/L or <50 × 10^9^/L in the first week of life, and found no differences in the incidence of new IVH or extension of existing IVH (the primary outcome) between the two groups (95). These results likely formed the basis for the use of 50 × 10^9^/L as the most frequent threshold for platelet transfusions in pre-term neonates.

The recently published much larger PlaNeT-2 multicenter trial randomized 660 thrombocytopenic neonates with a median gestational age of 26.6 weeks and a median birth weight of 740 grams to receive platelet transfusions at platelet count thresholds of <50 × 10^9^/L (<50 group) or <25 × 10^9^/L (<25 group). Unlike in the prior study, infants were randomized at any time during their NICU hospitalization when the platelet count fell below 50 × 10^9^/L, and the primary outcome was a composite of death or new major bleeding within 28 days of randomization (96). Ninety percent of infants in the <50 group and 53% in the <25 group received at least one platelet transfusion. Unexpectedly, infants in the <50 group had a significantly *higher* rate of mortality or major bleeding within 28 days of randomization compared to those in the <25 group (26 vs. 19%, respectively; odds ratio 1.57, 95%CI 1.06–2.32). In a subgroup analysis, findings were similar for neonates <28 weeks' gestation, the group at highest risk of bleeding and death (53, 55).

While these findings might have seemed surprising at first, they were in fact consistent with a number of prior observational studies describing a poor association between severity of thrombocytopenia and bleeding risk (51, 53–55), a lack of effectiveness of platelet transfusions to prevent bleeding in neonates (51, 97), and an association between number of platelet transfusions and neonatal mortality and morbidity (98–101).

The results of PlaNeT-2 provided high-level evidence in support of these concepts, although the possibility that the benefits of the lower transfusion threshold would be limited to clinically stable infants with a low risk of bleeding and/or death led to initial skepticism. This question was largely addressed in a follow-up study in which a multivariable logistic regression model was developed (as described above) (60) and used to predict the baseline bleeding/mortality risk of neonates enrolled in PlaNeT-2 (102). Based on their model-predicted baseline risk, 653 neonates in PlaNeT-2 were divided into four quartiles (very low, low, moderate, and high risk) and the absolute risk difference between the <50 group and the <25 group was assessed within each quartile. Interestingly, the lower transfusion threshold was associated with an absolute risk reduction in all four groups, varying from 4.9% in the lowest to 12.3% in the highest risk group. These results suggested that using a lower (<25 × 10^9^/L) prophylactic platelet transfusion threshold is beneficial even in high risk neonates (Table 1).

Although these studies provided strong support for the use of lower platelet transfusion thresholds in non-bleeding pre-term infants, some uncertainties remain. First, only 37% of infants in the study were randomized by day of life 5 and 59% by day 10, the period when most clinically significant hemorrhages occur in pre-term neonates (66). While this might have simply reflected the time of onset of thrombocytopenia in the study population, 39% of infants in PlaNeT-2 received one or more platelet transfusions prior to randomization, for unknown reasons and at non-specified platelet counts. This raises the question of whether these transfusions were given during the first few days of life, the highest risk period for IVH in pre-term neonates.

Nevertheless, the results of PlaNeT-2 provided strong support for the hypothesis that platelet transfusions may have deleterious effects in neonates, which could be mediated by various potential mechanisms. Neonates in PlaNeT-2, consistent with routine neonatal practices, were transfused with 15 mL/Kg of a platelet suspension. This is a substantially higher volume than that used in older children or adults, who usually receive ~5 mL/Kg of a standard platelet suspension. This high transfusion volume, combined with the fragile vasculature of pre-term neonates (see Intraventricular Hemorrhage above) and a rapid rate of transfusion, raises the possibility that the platelet transfusion itself could have caused or extended intraventricular hemorrhages through rapid volume expansion, thus providing a potential explanation for the higher incidence of major bleeding in the <50 compared to the <25 group. It has also been hypothesized that a “developmental mismatch” occurs when adult platelets are given to neonates. As reviewed above, adult platelets are functionally hyperreactive compared to neonatal platelets, and *in vitro* mixing studies found that adult platelets added to neonatal thrombocytopenic blood (with its higher hematocrit, MCV, and vWF levels) can induce a prothrombotic phenotype (103). Finally, it has become increasingly clear over the last decade that platelets have important functions beyond hemostasis, including as central mediators and modulators of inflammation (104, 105). Thus, it is plausible that some of the pathogenic effects of platelet transfusions on neonates could be mediated through inflammatory pathways. Additional work is needed to elucidate which of these potential mechanisms contribute to the increased mortality and morbidity associated with the liberal use of platelet transfusions in neonates, but in the meantime the data suggest that non-bleeding neonates (regardless of severity of illness) benefit from a restrictive platelet transfusion threshold.

### Fresh Frozen Plasma

Fresh frozen plasma (FFP) contains all of the clotting factors, fibrinogen, plasma proteins, electrolytes, protein C, protein S, antithrombin, and tissue factor pathway inhibitor. It is used primarily to replenish coagulation factors and is clinically indicated in the setting of hemorrhage, bleeding or severe coagulopathy due to multiple coagulation factor deficiencies secondary to liver disease, DIC, or congenital factor deficiencies for which there is no concentrate available (i.e., Factor V or XI deficiencies). While these are clear indications, for which FFP administration can be life-saving, FFP is most frequently given to non-bleeding neonates, especially those who are critically ill, either with the goal of preventing bleeding and/or for non-hematological indications (i.e., volume expansion in an infant with massive capillary leak). It has been estimated that 6–12% of all NICU admissions receive at least one FFP transfusion (106, 107), and in a study by Stanworth and colleagues 62% of infants who received FFP did not have signs of clinical bleeding at the time of transfusion, and 14% did not even have coagulation tests prior to FFP administration (108). Studies examining the success rate of FFP transfusion normalizing neonatal clotting times found success rates ranging from 40 to 60%, depending on the dose administered (107–109). The success rate of clotting time correction increased to 59–68% if neonatal reference ranges for coagulation factors were used (42). However, multiple studies have shown that, regardless of clotting time correction, administration of FFP does not change clinical outcomes. One study looking at FFP administration in the setting of DIC found no improvement in coagulation tests or in overall survival (110). Four studies have investigated the use of prophylactic FFP transfusion in pre-term neonates to decrease the incidence of IVH: One found a decrease in IVH with FFP administration, but the other three reported similar IVH rates in their control and treatment arms (111–114) and a meta-analysis found no differences in grade of IVH or mortality (115) (Table 3). Additional controlled studies have found no benefit of FFP administration to non-bleeding neonates with sepsis, respiratory distress syndrome, hypotension, or hypoxic ischemic encephalopathy (116–120). Despite this growing evidence, FFP continues to be administered to non-bleeding neonates outside of the evidence-based recommendations, in many instances in response to “prolonged” coagulation tests that might be developmentally appropriate.

### Recombinant FVIIa and Prothrombin Complex Concentrate

Recombinant Factor VIIa (rFVIIa) is a genetically engineered coagulation protein initially created for the treatment of bleeding in patients with hemophilia and antibodies against standard coagulation factor replacements. It is extremely effective in activating the final pathway of the coagulation cascade and has been perceived by some as a “universal hemostatic agent,” prompting its frequent off-label use in bleeding patients without hemophilia. Since bleeding and coagulation disorders are common in neonates, rFVIIa is an attractive solution to an unsolved problem. Several case reports have described the successful administration of rFVIIa to pre-term and term neonates with intractable hemorrhage and/or severe coagulopathy (121). One retrospective report of 29 neonates found that earlier rFVIIa administration (<24 h from beginning of bleeding) was associated with a statistically significant improvement in survival (121) and with a decreased need for subsequent blood products. However, concerns have been raised regarding the safety profile of rFVIIa, specifically a potential increase in thrombotic events (122). A systematic review of neonates receiving rFVIIa or FFP found no difference in the occurrence of thrombotic events in neonates with bleeding or coagulopathy (123). However, while multiple randomized controlled trials of rFVIIa administration have been performed in adults, only three studies have included children with a total of 11 neonates, and so high-quality data is lacking to guide the safe use of rFVIIa in this population and concerns over increased risk of thrombosis remain.

Prothrombin complex concentrate (PCC) is a human plasma-derived product containing the vitamin K-dependent coagulation factors and the vitamin K-dependent clotting inhibitor proteins. The current indications for PCC are the treatment and perioperative prophylaxis of bleeding in congenital or acquired deficiency of prothrombin complex coagulation factors. Increasingly, it has been used off-label in hopes of preventing and treating severe bleeding in neonates. In 2002, The Guideline for the Investigation and Management of Neonatal Hemostasis and Thrombosis recommended considering PCC when high volume products (such as FFP) need to be avoided or in the presence of hemorrhage due to depleted factors (124). Due to its smaller volume, PCC can be infused quicker than, and corrects the INR faster than FFP (41 vs. 115 min) (124, 125). As with rFVIIa, there are no randomized controlled studies to guide the safe use of PCC in the neonatal population, but a recent retrospective study examined 37 neonates with intractable bleeding or severe coagulopathy who received PCC as a rescue intervention. In this study, hemostasis was achieved in the majority of infants and there was a statistically significant improvement in PT, INR, and PTT. Thirteen out of 24 neonates survived. PCC had been administered to the neonates who survived within 24 h of bleeding initiation and no thrombotic events were observed (126). As with rFVIIa, randomized controlled trials or prospective controlled studies are needed to determine the efficacy and safety of PCC in the neonatal population before it can become part of the standard care of a bleeding neonate.

## Conclusions

The neonatal hemostatic system is strikingly different from that of adults in that neonates exhibit comparatively hyporeactive platelets and decreased levels of coagulation factors, the latter translating into prolonged clotting times (PT and PTT). Since pre-term neonates have a high incidence of bleeding, particularly IVH, neonatologists frequently administer blood products (i.e., platelets and FFP) based on arbitrary laboratory thresholds in an attempt to overcome these “deficiencies” and reduce the bleeding risk. However, it has become increasingly clear that both the platelet hyporeactivity as well as the decreased coagulation factor levels are effectively counteracted by other factors in neonatal blood that promote hemostasis (i.e., high levels of vWF, high hematocrit and MCV, reduced levels of natural anticoagulants), resulting in a well-balanced neonatal hemostatic system, perhaps slightly tilted toward a prothrombotic phenotype (Figure 1). While life-saving in the presence of active major bleeding, the administration of platelets and/or FFP to non-bleeding neonates based on laboratory tests has not only failed to decrease bleeding, but has been associated with increased neonatal bleeding and mortality in the case of platelets. Given the unique features of neonatal hemostasis, there has been interest in exploring the potential use of new tests of whole blood hemostasis (i.e., TEG or ROTEM) or primary hemostasis (i.e., PFA-100 CT-ADP) to predict and/or manage bleeding in neonates. However, more studies are needed to establish the potential value of these tests in the management of neonates of different gestational ages and with different clinical conditions. With an increased understanding of neonatal hemostasis and *in vivo* factors that increase a neonate's bleeding risk, it might be possible to develop novel and more accurate approaches to manage the hemostatic challenges of critically ill neonates.

## Author Contributions

PD and MS-V reviewed the literature, wrote, and edited the manuscript. Both authors contributed to the article and approved the submitted version.

## Conflict of Interest

The authors declare that the research was conducted in the absence of any commercial or financial relationships that could be construed as a potential conflict of interest.
